# In Play We Trust. Rapid Facial Mimicry Predicts the Duration of Playful Interactions in Geladas

**DOI:** 10.1371/journal.pone.0066481

**Published:** 2013-06-13

**Authors:** Giada Mancini, Pier Francesco Ferrari, Elisabetta Palagi

**Affiliations:** 1 Dipartimento di Biologia Evolutiva e Funzionale, Università di Parma, Parma, Italy; 2 Museo di Storia Naturale, Università di Pisa, Calci, Pisa, Italy; 3 Istituto di Scienze e Tecnologie della Cognizione, CNR, Roma, Italy; University of Tasmania, Australia

## Abstract

The primate play-face is homologous to the human facial display accompanying laughter. Through facial mimicry, the play-face evokes in the perceiver a similar positive emotional state. This sensorimotor and emotional sharing can be adaptive, as it allows individuals to fine-tune their own motor sequences accordingly thus increasing cooperation in play. It has been recently demonstrated that, not only humans and apes, but also geladas are able to mimic others' facial expressions. Here, we describe two forms of facial mimicry in *Theropithecus gelada*: rapid (RFM, within 1.0 s) and delayed (DFM, within 5.0 s). Play interactions characterized by the presence of RFM were longer than those with DFM thus suggesting that RFM is a good indicator of the quality of communicative exchanges and behavioral coordination. These findings agree with the proposal of a mirror mechanism operating during perception and imitation of facial expressions. In an evolutionary perspective, our findings suggest that RFM not only was already present in the common ancestor of cercopitecoids and hominoids, but also that there is a relationship between RFM and length and quality of playful interactions.

## Introduction

Facial displays regulate many aspects of social life such as aggression, dominance-subordinate relationships, social affiliation, appeasement, and play [1]–[5]. Play is an excellent field to examine the role of signals in emotional/intentional communication systems [6]. Indeed, through play animals acquire the ability to regulate their emotional responses [7] and this, in turn, affects the development of skills to perform actions and facial expressions in the appropriate context, thus increasing social competence [7]. The play-face in non-human primates is homologous to the human facial display accompanying laughter [8], [9]. The play face perception often induces in the observer the activation of the same motor programs. In humans, this phenomenon named facial mimicry, evokes in the perceiver not only a similar facial expression but also the corresponding positive emotional state (emotional resonance) [10], [11].The ability to instantly understand others' emotional states allows an individual to foresee playmates' intentions [6] and fine-tune its own motor sequences accordingly [11]. In this view, such sensorimotor and emotional sharing is a prerequisite to avoid any misunderstanding, manage a playful interaction successfully, promote social affiliation, and increase cooperation levels [6], [7], [12]–[14].

Facial mimicry is an involuntary, rapid, and automatic response, in which an individual mimics the facial expression of another individual. This phenomenon can be distinguished from other forms of imitation [15], [16] due to the rapidity of the response (Rapid Facial Mimicry, RFM) involving exclusively the face. People mimic emotional facial expressions of others within 1,000 ms [17]. RFM has been widely described in children [18], [19] and adult humans, *Homo sapiens* [20], whose mirroring reactions are elicited more frequently and rapidly in response to a dynamic facial expression compared to a static one [21]. More recently this phenomenon has been described also in orangutans [22], chimpanzees [12] and geladas [23].

If RFM represents an important facial response which helps the players to synchronize their bodily motor actions and to improve the success of the playful interaction, it is expected that the presence of RFM, and not of other, non-matched facial responses, correlates with the quality and duration of playful interactions.

Facial mimicry responses have been analyzed at a finer scale according to the speed of the response. In particular, two time domains have been identified to describe mimicry responses of human positive facial expressions (smile and laughter): 1) response occurring within 1.0 s after the perception of the stimulus and 2) response occurring between1.0 and 5.0 sec [17], [20]. It has been suggested that these two types of responses are not part of the same continuum, but they could be qualitatively different and, therefore, likely reflecting a partial differential modulation from the underlying neural substrates [24]. The first type, named Rapid Facial Mimicry (RFM), could be related to the spontaneous human Duchenne laughter, while the second type, named Delayed Facial Mimicry (DFM), could be connected with the non-Duchenne laughter. This latter form of mimicry has been proposed to be a non-automatic response [1]. It is probably under voluntary control and likely detached from emotions [23]–[25].

It has been recently observed that also chimpanzees can produce mimic responses that are rapid or delayed [12]. However, these two responses do not produce different effects on the quality and duration of the interaction [12], thus casting doubts on the dichotomous nature of these two forms of facial mimicry in terms of both functions and underlying mechanisms.

Mancini and colleagues [23] provided evidence that RFM occurs also in a non-ape species, the gelada (*Theropithecus gelada*), and that both immature and adult subjects mimicked within 1 sec the play faces of others. More interestingly, the results showed that the latency of RFM varied across the different dyads. Moreover, the speed and rates of RFM were correlated with the quality of bonding characterizing each dyad (mother-offspring *versus* mother-non offspring) [11], [23]. These findings suggest that in geladas RFM can raise in the perceiver a strong emotional positive response which can predict the quality of social play interactions. Geladas could be therefore a good model to test whether different forms of facial mimicry, based on the accuracy and speed of facial response, are related to playful interaction quality, measured by the duration of play bouts. Here, we test this hypothesis by characterizing the response accuracy (matched *versus* non-matched) and speed of facial mimicry (RFM and DFM). We predict that longer playful interactions are accompanied by 1) higher frequency of matched (mimicry) compared to non-matched facial responses and 2) higher frequency of RFM compared to DFM events.

## Methods

### Ethics statement

This study was approved by University of Pisa and Parma (Animal Care and Use board). Since the study was purely observational the committee waived the need for a permit. The study was conducted with no manipulation of animals.

### The colony and the data collection

The colony of geladas (Rheine, Germany) was composed of two one-male units (OMUs), which included two adult males, 18 adult females, and 18 immature subjects. Kin relations were known. The OMUs lived in enclosures both with indoor (36 m^2^) and outdoor facilities (2,700 m^2^). Animals were fed two times a day (9:30 a.m., 2:30 p.m.) with water available *ad libitum*. No stereotypic/aberrant behaviors have been ever observed. The research complies with current laws of European Community.

We focused our analysis on rough and tumble, the most common form of social play in this species [26]. Dyadic play bouts (n = 1,121) were video-recorded during a 6-month period (2009–2010). A play bout began when one partner directed any playful pattern toward a conspecific and ended when playmates ceased their activities, one of them moved away, or when a third individual interrupted the interaction. If a play session started after a delay of 10 s it was counted as a new session.

We defined RFM as the visible response of facial musculature by an observer to match the facial gestures in another individual's facial expression. This congruent response must be rapid: within 1 sec from the emission of the stimulus. We focused our analysis on two expressions: PF (play-face, mouth opened with only the lower teeth exposed) and FPF (full play-face, lower/upper teeth and gums exposed via the active retraction of the upper lip). During play, geladas frequently lip smacked (LS, lips are protruded and then smacked together repeatedly, sometimes alternated with tongue protrusions) toward conspecifics. LS, a non-context specific expression, is a facial display used to signal benign intentions [11].

Video analysis was conducted via Kinovea v. 0.7.10. Videometric analyses were mainly conducted by G.M. with the help of E.P. Interobserver reliability was tested with one frame/4 msec accuracy. The mean Cohen's kappa values were 0.78 for PF, 0.81 for FPF, and 0.76 for LS.

To be reasonably sure that the expression performed by the observer was actually elicited by the trigger's expression, we considered only those interactions where the observer looked at the face of the trigger and did not show any expression 1 s prior to the trigger's stimulus. Chewing/biting transitional faces were excluded to reduce uncertainties. Each play session could involve more than one facial triggering event. In this case, we considered as new the subsequent triggering event that occurred after the two playmates had interrupted the visual contact for at least 2 sec.

We distinguished play interactions characterized by: i) *no facial expressions* (absence of PF/FPF), ii) *no facial response* (PF/FPF perceived without replication in 75% of cases), iii) *incongruent facial response* (at least 75% of facial responses were incongruent: PF or FPF as stimulus and LS as response), and iv) *congruent facial response* (at least 75% of facial responses were congruent: PF stimulus/PF response, FPF stimulus/FPF response).

Following the criteria used for human studies [17], [27], the facial responses were also measured in two time domains: within the first second after the onset of a facial stimulus emitted by a playmate (rapid mimicry: <1 sec) and within the following 4 sec (delayed mimicry: >1 sec and < 5 sec). Considering the two time domains and the congruence we distinguished four types of play sessions according to the 75% criterion: i) *incongruent delayed facial response* (>1 sec and <5 sec), ii) *incongruent rapid facial response* (<1 sec), iii) *delayed facial mimicry* (>1 sec and <5 sec), and iv) *rapid facial mimicry* (<1 sec).

We calculated the frequency of the different responses as a function of the stimulus events perceived for each individual. This made possible to collect more than one triggering event for each observed individual. Due to subject variability in terms of play frequency and facial expressions, the analysis was carried out at an individual level for a more conservative statistical approach. We restricted the analysis to those subjects, who showed all the possible response combinations (N = 20 in the first set of analyses; N = 14 in the second set of analyses).

Due to non-normal data distribution, we employed nonparametric statistics and exact tests. To compare the individual mean length of the sessions we applied the Friedman test. We employed the Dunnett's multiple comparison test to determine what pairs of playful interactions significantly differed.

## Results

**Figure 1:**
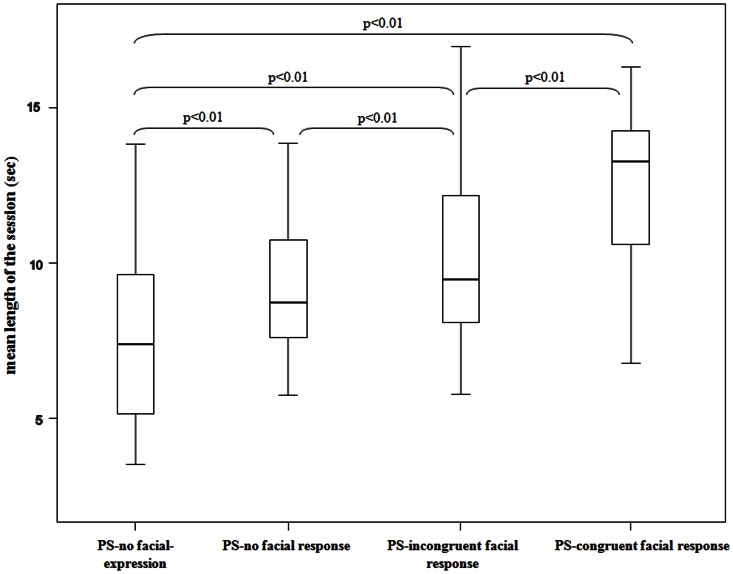
Duration lengths of play sessions (PS) characterized by 1) no facial expressions, 2) no facial response, 3) incongruent facial response, and 4) congruent facial response.

The play duration length significantly differed across the conditions: no facial expressions, no facial response, incongruent facial response, and congruent facial response (Friedman's *χ^2^* = 19.800, *N* = 20, *df* = 3, *p*<0.001). The Dunnett's test revealed that sessions characterized by congruent response were longer than those with no facial expressions (*q* = 4.41; *p*<0.01), facial expressions without response (*q* = 2.84; *p*<0.01), and incongruent response (*q* = 3.13; *p*<0.01). Moreover, sessions characterized by the presence of PF/FPF without any response were longer than those sessions characterized by the absence of any facial expression (*q* = 4.02; *p*<0.01), but did not significantly differ from incongruent response sessions (*q* = 0.89; *p*>0.05) (Figure 1).

**Figure 2:**
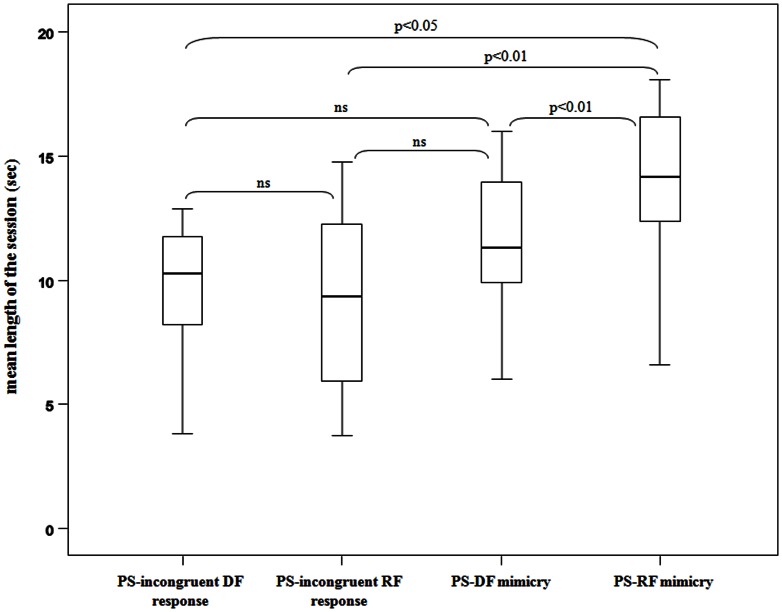
Duration lengths of play sessions (PS) characterized by 1) incongruent delayed facial (DF) response, 2) incongruent rapid facial (RF) response, 3) delayed facial (DF) mimicry, and rapid facial (RF) mimicry.

The duration lengths of play interactions characterized by incongruent delayed response, incongruent rapid response, DFM, and RFM significantly differed (Friedman's *χ^2^* = 10.079, *N* = 14, *df* = 3, *p* = 0.015). The Dunnett's test revealed that sessions characterized by the presence of RFM were longer than those with DFM (*q* = 4.27; *p*<0.01), those characterized by the presence of incongruent delayed response (*q* = 2.01; *p*<0.05) and incongruent rapid response (*q* = 3.86; *p*<0.01). Finally, no difference in play duration length was found between the other session's conditions: incongruent delayed response *vs* incongruent rapid response (*q* = 1.86; *p*>0.05); incongruent rapid response *vs* DFM (*q* = 1.20; *p*>0.05) and incongruent delayed response vs DFM (*q* = 0.03; *p*>0.05) (Figure 2).

## Discussion

In geladas, the playful interactions characterized by a higher frequency of facial mimicry had also a longer duration. This suggests that the effectiveness of a facial expression is amplified when it is matched by the observer. Compared to the mere perception of a playful facial display not followed by the matched response (Figure 1 and 2), facial mimicry appears to convey more important information to the playmate. It signals not only that the stimulus has been perceived but that it has been accurately interpreted. This pattern of facial interaction limits possible ambiguity generated by the lack of response or by an incongruent response that may communicate a non clear interpretation of the signal by the perceiver. Thus, facial mimicry might facilitate communicative exchanges and behavioral coordination in the following sequence of actions. Being able to prolong the interaction is advantageous for the playmates who increase the opportunity to assess their reciprocal ability and to test their social relationship. Indeed, play is one of the best tools which leads individuals to improve social competence [7], reinforce social bonds [26], and learn how to manage tension situations [11].

In geladas, the climax in the play duration length was reached when mimicry was rapid, thus suggesting that the automatic response is more involved in the modulation of playful interactions than the delayed response. Although internal and external factors can delay the mimicry response, it is possible that DFM is more strongly modulated by internal factors that exert a stronger suppression of the motor output compared to RFM. In contrast, RFM reveals an automatic nature, probably as the consequence of a stronger input from emotional networks.This finding is in line with the studies in humans showing that the RFM and DFM reflect different levels of voluntary control and of the neurophysiological mechanism controlling them [17], [20], [27], [28]. In this perspective, our results in geladas support the idea of an evolutionary continuum in the emotional and behavioral processes regulating affiliative interactions during play behavior in primates.

These data also raise important questions concerning the cause-effect relationship between the processing of facial expressions and the associated behavioral output. In other terms, is RFM a byproduct of a high motivational state to play (and to play longer), or does RFM stimulate longer playful sessions? From the current findings we cannot disentangle which is the cause-effect relationship between RFM and the real emotional engagement to play. However, it is worth noting that when the response is rapid (reflecting a high arousal state) but not congruent, the play bout length is shorter than when the response is rapid and congruent (Figure 1). This suggests that RFM *per se* could act as a trigger to prolong play, even though further studies are needed to assess the cause-effect relation between RFM and the emotional state of the player.

From a neurobiological perspective, our findings also support the mirror neuron hypothesis [4], [5], [29], [30] according to which the observation of an emotional facial expression activates in the subjects corresponding facial motor representations [31]. The observation of a facial expression activates not only the motor programs involved in the production of the same display, but also the same internal emotional state associated to it [32]–[34]. The shared emotional/sensorimotor representation might have an impact in promoting a sense of familiarity between two individuals, thus facilitating the emotional connection that can be generated during RFM.
